# The asbestos paradox: global gaps in the translational science of disease prevention

**DOI:** 10.2471/BLT.14.142307

**Published:** 2015-02-27

**Authors:** Oladele A Ogunseitan

**Affiliations:** a Department of Population Health and Disease Prevention, Program in Public Health, 653 East Peltason Drive, University of California, Irvine, California, 92697-3957, United States of America.

Mesothelioma, a malignant cancer of the pleura, is almost exclusively associated with asbestos exposure.^1^ What will it take to eradicate this highly preventable cancer? Despite the scientific consensus that exposure to asbestos fibres causes deadly diseases, we continue to use asbestos in products designed ostensibly to improve quality of life. For example, asbestos is still used in automobile brake-linings, and in the chloralkali industry that produces chlorine for disinfecting water worldwide.^2^ Toxic asbestos remains the main material for some plastics and in domestic building products – especially in corrugated roof materials for housing in developing countries.

Regional disparities persist in translating scientific knowledge of asbestos risks to policy for preventing cancers and other diseases. Most cases of mesothelioma are now found in countries producing asbestos and in developing countries using the products, where scientific knowledge of asbestos toxicity seems to have been lost in translation.^3^ It is in these countries, where affected populations are less likely to have access to prompt diagnosis, health care or litigation, that we will observe the next wave of mesothelioma cases.^3^ A perspective on three scientific translational gaps is presented here: (i) making policy decisions within the context of scientific uncertainty, (ii) the role of alternative assessments in selecting safer commercial materials, and (iii) the translation of scientific evidence into disease prevention.

**Fig. 1:**
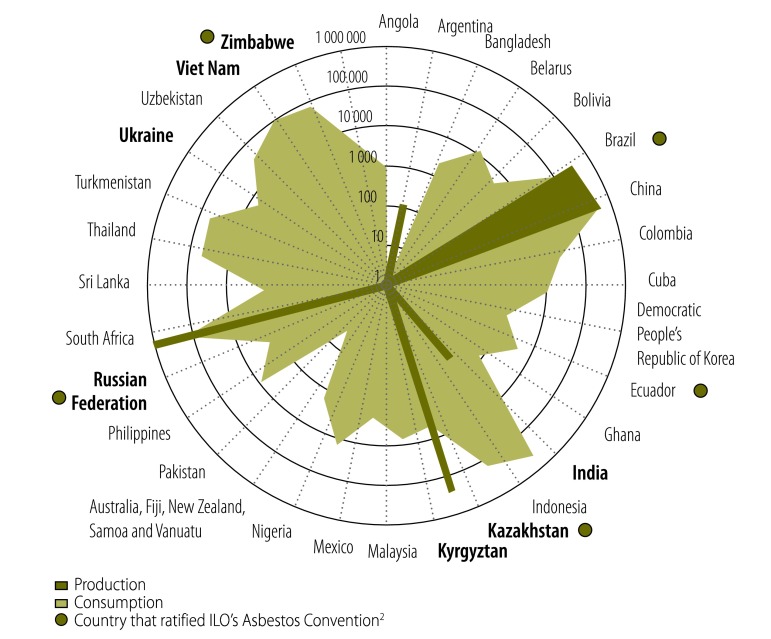
Asbestos producers and consumers, 35 countries, 2011

Notes: ILO: International Labour Organization. Seven countries, written in bold typeface, voted to block the initiative to require prior informed consent for international shipment of chrysotile asbestos during the 2013 Conference of Parties to the Rotterdam Convention.

The International Labour Organization’s Asbestos Convention, designed to protect workers from the well-known hazards of asbestos exposure, entered into force on 16 June 1989. Yet, nearly twenty-five years later, only 35 countries, 19% of 184 that are eligible, have formally ratified the Convention.^2^ In contrast, 154 countries (83% of those eligible) are Parties to the 1998 Rotterdam Convention on the Prior Informed Consent Procedure for Certain Hazardous Chemicals and Pesticides in International Trade.^4^ However, attempts to include chrysotile asbestos – the most common form used – in Annex III of the Rotterdam Convention have failed repeatedly, most recently at the sixth Conference of Parties due to resistance from seven countries, most of whom continue to produce, use, and export asbestos on a large scale (Fig. 1). The minority objection prevailed despite powerful testimony from the representative of the World Health Organization (WHO) that chrysotile and all forms of asbestos are carcinogenic to humans.^6^ Moreover, given the widespread use of chrysotile in domestic building products, it is impossible to safeguard hazardous exposures in occupational settings or to prevent environmental contamination that threatens the general population. Opposition to the proposal to regulate chrysotile asbestos under the Rotterdam Convention highlights three major gaps in the translation of scientific evidence to global disease prevention policy.

Data sources: Rotterdam Convention on the Prior Informed Consent Procedure for Certain Hazardous Chemicals and Pesticides in International Trade UNEP/FAO/TC/COP.6/20^4^and asbestos statistics and information.^5^

The first gap concerns making policy decisions within the context of scientific uncertainty. For example, the representative of one country claimed that there are insufficient scientific data to support listing asbestos under the Rotterdam Convention. This objection reflects a lack of understanding of the complexities of the scientific process and the inherent variability of factors that contribute to the relationship between toxic exposures and disease outcomes. There are small differences in the findings of observational studies of the effects of asbestos exposure. These are the result of complex interactions between asbestos exposure and genetic, environmental and social factors. Published research in this field includes studies contributed by laboratories that may have a conflict of interest with the asbestos industry.^7^ This complicates any assessment of the scientific evidence. In practice, a combination of different categories of evidence are used to reach policy decisions that protect the most vulnerable people.^8^ The proposed requirement of informed consent for the use of chrysotile is based on consistent and reliable evidence from numerous empirical and mechanistic studies conducted by independent scientists.

The second gap is the role of alternatives assessments in the selection of safer materials used in consumer products. For example, at least two representatives of countries opposing the international regulation of chrysotile asbestos claim that alternative materials have not been sufficiently researched to compare the risks to human health and the environment. Also, claims have been made that technical assistance is lacking for countries wishing to phase out asbestos use and replace it with safer alternatives. The scientific methods developed to address this issue – including materials’ life cycle assessment and functional equivalence – struggle with making comparisons across very different characteristics and potential health and environmental impacts. The assessment methods also have to compare a range of plausible disease endpoints. Whereas a particular material used in a consumer product may be linked to respiratory diseases, proposed alternatives may be linked to reproductive health effects, cognitive deficits or to cancers.^9^ Although there are legitimate scientific issues to be resolved with alternative assessments, they do not amount, in the case of asbestos, to reasons for stalling policy procedures that simply require informed consent. Inclusion of asbestos under the Rotterdam Convention would increase the pace of research to discover safer alternative materials and their adoption by key industries.

The third gap in the efficient translation of scientific evidence to disease prevention policy is the need to acknowledge trade-offs between public health, industrial development, economic advancement, employment and political autonomy. Representatives of countries opposing the listing of asbestos under the Rotterdam Convention acknowledge that this inclusion would not imply a ban on production, but argue that it would have a negative impact on international trade and contribute to unemployment. Without political consensus, regulations may be ineffective as detractors identify and use loopholes and backdoor trading to circumvent international policy. This has been the case, for example, with toxic electronic waste and the Basel Convention for controlling transboundary movements and disposal of hazardous wastes.^10^^,^^11^ Yet, forward-thinking countries manage to withstand pressure to protect economic interests at the expense of public health. This is exemplified by the implementation of California’s landmark safer consumer products regulations after five years of contemplation and debate surrounding these trade-offs.^12^

It is possible to eradicate mesothelioma, but we must work harder to bridge the gaps between scientific knowledge and policy decisions that should protect people.
